# Observation of image pair creation and annihilation from superluminal scattering sources

**DOI:** 10.1126/sciadv.1501691

**Published:** 2016-04-15

**Authors:** Matteo Clerici, Gabriel C. Spalding, Ryan Warburton, Ashley Lyons, Constantin Aniculaesei, Joseph M. Richards, Jonathan Leach, Robert Henderson, Daniele Faccio

**Affiliations:** 1 School of Engineering, University of Glasgow, Glasgow G12 8LT, UK.; 2 School of Engineering and Physical Sciences, SUPA, Heriot-Watt University, Edinburgh EH14 4AS, UK.; 3 Department of Physics, Illinois Wesleyan University, Bloomington, IL 61701, USA.; 4 Institute for Micro and Nano Systems, University of Edinburgh, Alexander Crum Brown Road, Edinburgh EH9 3FF, UK.

**Keywords:** Physics, optics, speed of light

## Abstract

The invariance of the speed of light is one of the foundational pillars of our current understanding of the universe. It implies a series of consequences related to our perception of simultaneity and, ultimately, of time itself. Whereas these consequences are experimentally well studied in the case of subluminal motion, the kinematics of superluminal motion lack direct evidence or even a clear experimental approach. We investigate kinematic effects associated with the superluminal motion of a light source. By using high-temporal-resolution imaging techniques, we directly demonstrate that if the source approaches an observer at superluminal speeds, the temporal ordering of events is inverted and its image appears to propagate backward. Moreover, for a source changing its speed and crossing the interface between subluminal and superluminal propagation regions, we observe image pair annihilation and creation, depending on the crossing direction. These results are very general and show that, regardless of the emitter speed, it is not possible to unambiguously determine the kinematics of an event from imaging and time-resolved measurements alone. This has implications not only for light, but also, for example, for sound and other wave phenomena.

## Teaser

Researchers experimentally investigate the superluminal kinematics of scattering light sources through ultrafast time-resolved imaging.

## INTRODUCTION

In a display of prescient intuition, Lord Rayleigh noted that a supersonic source of sound waves could give rise to time reversal of the perceived sound by a stationary observer. For the specific one-dimensional case in which the source moves at exactly twice the speed of sound, “sounds previously excited would be gradually overtaken and heard in reverse of natural order… the observer would hear a musical piece in correct time and tune, but *backwards*” (*1*). Unfortunately, any attempt to actually play out such an experiment is faced with wave attenuation over the huge distances (~1 km) covered by a supersonic source while emitting just 3 s of music. However, the reasoning followed by Lord Rayleigh solely relies on the fact that the wave speed is finite and independent of the speed of the emitter. Therefore, the same result also holds true for light waves.

Contrary to typical expectations, it is possible to create a superluminal source of light, where we use the term “source” in a very broad sense. Consider, for example, a wavefront impinging on a flat surface such as a wall: The intersection point of the wavefront with the wall moves at a speed *v* = *c*/sinθ, where *c* is the speed of light in vacuum and θ is the angle made between the vector orthogonal to the wall surface and the wave vector. Therefore, *v* > *c* for all wavefront propagation angles. Moreover, this intersection point will, in general, always be visible owing to scattering from the wall surface. Thus, although there is no physical source of light moving at *v* > *c*, we nevertheless have a superluminal “scattering source” that can be used to study and observe the kinematics of superluminal phenomena.

Superluminal sources, or more precisely sources with a group velocity exceeding the vacuum speed of light *c*, were given a precise description by Brillouin (*2*, *3*) and then observed in a number of different optical arrangements, for example, in “fast-light” media (*4*), the propagation of Bessel beams (*5*–*9*), and Lyot filters (*10*), and from scattering surfaces (*11*). Although it is now accepted that superluminal group velocity does not contradict the theory of special relativity because the information speed is always limited by *c* [see, for example, the study of Stenner *et al.* (*12*)], to our knowledge, time ordering or, in general, the kinematics associated with superluminal speeds has not yet been experimentally addressed, and there has been no prior demonstration of the image pair creation and annihilation shown in this work.

Here, we present a series of experiments that rely on ultrafast imaging techniques, which illustrate various kinematic phenomena, including time reversal and image pair creation and annihilation at transitions from subluminal to superluminal propagation.

## RESULTS

**Fig. 1:**
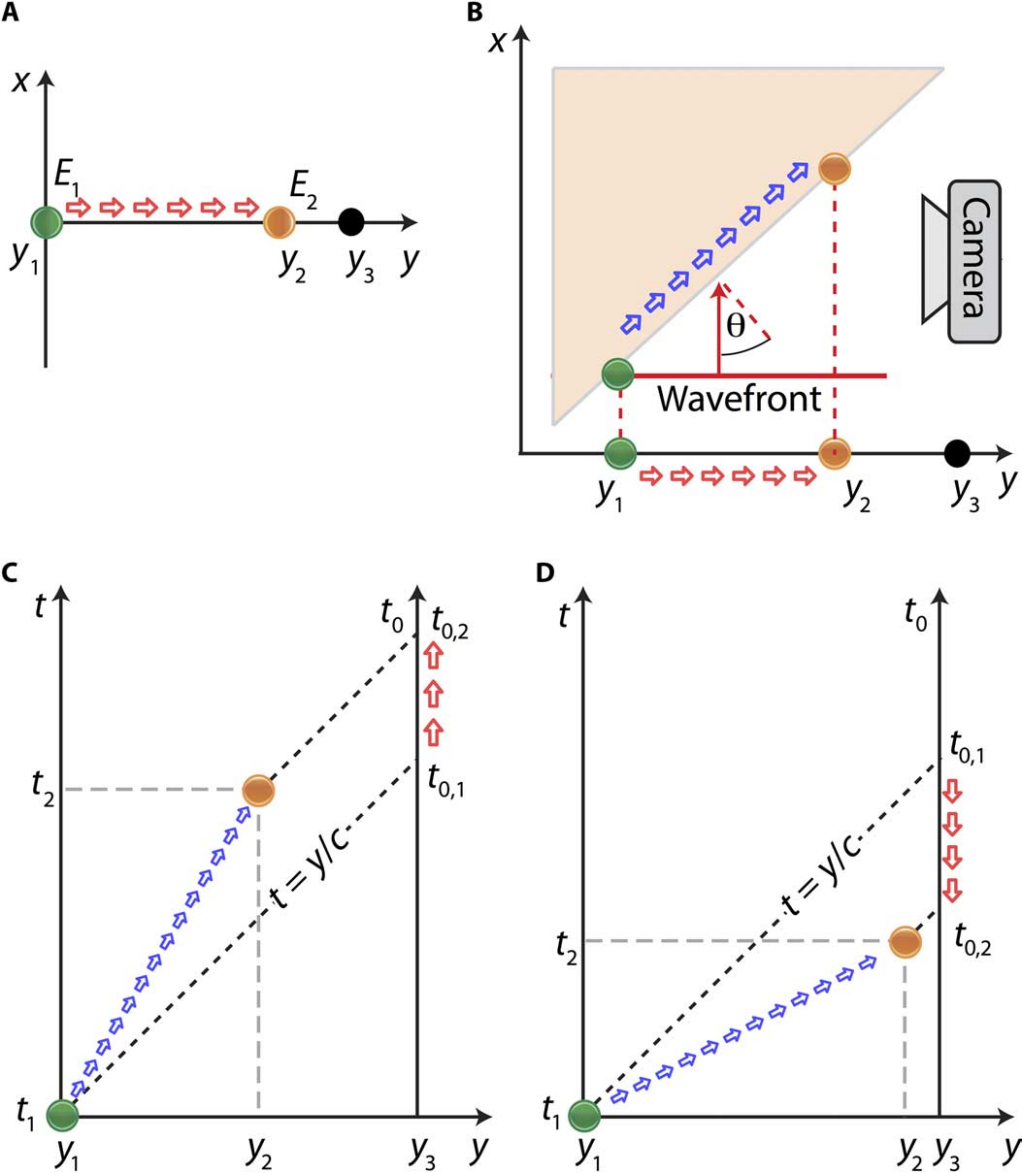
Space-time diagrams. (**A**) An illustration of the (1 + 1)*D* example described in the text. (**B**) The extension of (A) to a (2 + 1)*D* case that exemplifies the experimental layout. The motion of the scattering source toward the observer (red arrows) can be either superluminal or subluminal. (**C**) Minkowski diagram for two sequential events. Because this case has *v < c*, time ordering is preserved. (**D**) Minkowski diagram for two (causally disconnected) events where *v* > *c*: time ordering is inverted.

For the purpose of illustration, we first consider the simplest (1 + 1)*D* (one spatial and one temporal dimension) situation sketched in Fig. 1A where we consider two events, *E*_1_(*y*_1_, *t*_1_) and *E*_2_(*y*_2_, *t*_2_), taking place at two separate positions (*y*_1_, *y*_2_) and times (*t*_1_, *t*_2_), associated with a moving source. We also consider an observer with a camera in a fixed reference frame identified as the laboratory frame and at a position *y*_3_. Whereas the original time delay between *E*_2_ and *E*_1_ is Δ*t* = *t*_2_ − *t*_1_, the time difference recorded by the observer (at position *y*_3_) is obtained considering that the information of these events travels to the camera at the speed of light *c*

$$\Delta t_{\text{observer}}=(t_{2}+\frac{y_{3}-y_{2}}{c})-(t_{1}+\frac{y_{3}-y_{1}}{c})=\Delta t(1-\frac{v}{c})$$ (1)

where *v* is the speed of the source along the *y* direction. For *v* < *c*, the observer will perceive a reduced time delay, but the time ordering of the events is preserved. However, if *v* > *c*, the time ordering will be inverted as Δ*t*_observer_ < 0. In other words, if the observer is trying to record the image of a superluminal object, then they will not be able to tell from the time-resolved video data alone whether the source is approaching or moving away from them.

The geometry we investigate in our experiments (Fig. 1B) is that of a superluminal spot (blue arrows) created by a pulsed plane wave (shown with a red line), which is itself propagating at the speed of light and impinging on a tilted screen. As already noted, this spot propagates at speeds that are superluminal relative to the screen, regardless of the screen angle. However, the component of the velocity of the spot along the direction toward the observer (here, along the *y* direction) will, in general, depend on the screen angle. Therefore, this latter velocity can be experimentally tuned from subluminal to superluminal by simply tuning the screen inclination angle.

The perceived temporal inversion of events relies only on two ingredients: The wave speed should be finite and independent of the emitter speed, and the emitter should be moving faster than the wave speed in the direction of the observer. The generality of these conditions can be seen by plotting the kinematics in the relative Minkowski space-time diagrams for the subluminal (Fig. 1C) and superluminal cases (Fig. 1D). In the subluminal case, the worldline of the emitter (blue arrows) lies above the lightline $\left(t=\frac{y}{c}\right)$, and the measured arrival times of these emanations, for a stationary observer at *y*_3_, retain proper time ordering (indicated by the red arrows). Conversely, a superluminal emitter’s worldline lies in the region below the lightline. Geometrical construction of the stationary observer’s measurements of the same events shows that these must be characterized by a time-ordering inversion (indicated by the downward orientation of the red arrows in Fig. 1D).

We stress that although superluminal motion is involved here, there is no superluminal transfer of information because the scattering events at distinct regions of the screen are not causally connected (they belong to physically distinct regions of the incoming wavefront). Moreover, we do not need to consider relativistic effects or Doppler shifts because there are no dipole emitters that are actually moving.

For the geometry established in Fig. 1B, the component of the spot velocity in the *y* direction of the observer is simply given by *v* = *c* cotθ. Therefore, the component of the spot velocity in the direction of the observer is superluminal for 0 < θ < π/4 and subluminal for π/4 < θ < π/2. Following the considerations of Eq. 1, the observer will record an inverted time order of the events for the former case. Straightforward generalization of this argument reveals that time-ordering inversion results whenever the angle of detection is greater than the angle of incidence. Furthermore, the inverted time ordering also modifies the observer’s perception of the speed of the scattering source along the $\hat{x}$ direction. Indeed, the recorded speed along $\hat{x}$ is

$$v_{x}^{0}=\frac{dx}{dt_{0}(x)}=\frac{c}{1-\text{cot}\theta }$$ (2)

where the time *t*_0_(*x*) is the arrival time of the signal on the *x* position of the detector (see the Supplementary Materials).

Therefore, for 0 < θ < π/4, the perceived speed along the $\hat{x}$ direction has an opposite sign with respect to the real speed. In such circumstances, even a detector with sufficient resolution to track the events will not be able to distinguish between a source moving from left to right at superluminal speed from one moving in the opposite direction at subluminal speed.

**Fig. 2:**
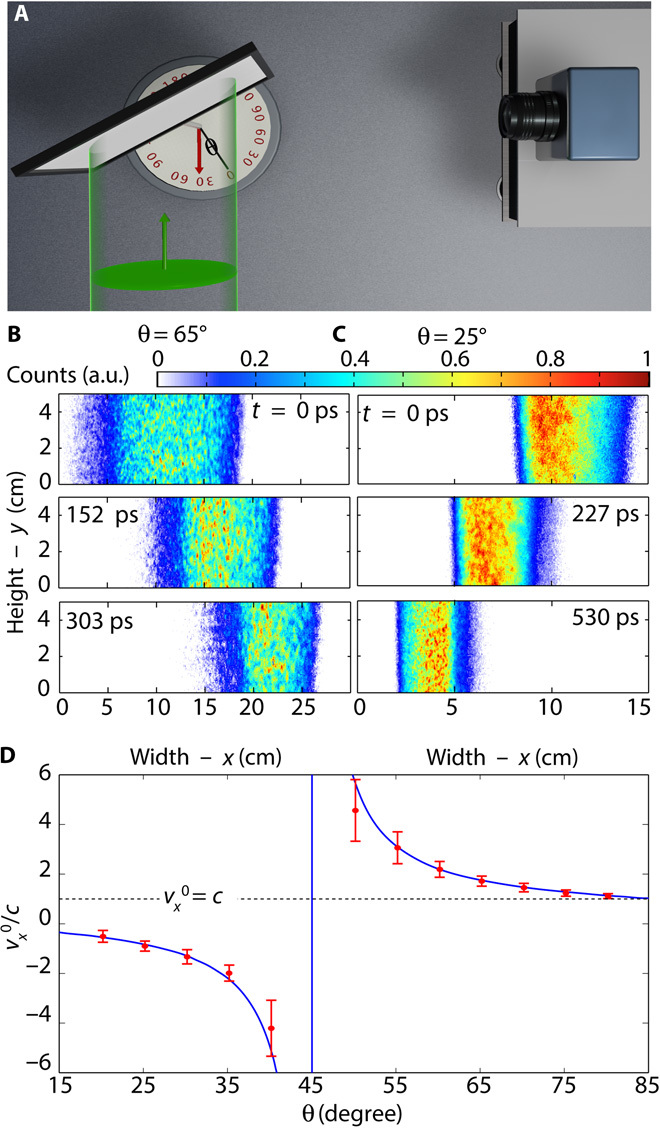
Time order inversion. (**A**) A sketch of the experiment. A plane wavefront (green) impinges on a tilted screen, and the scattered radiation is recorded at 90° with a time-resolving intensified charge-coupled device (iCCD) camera. Changing the angle θ between the input wave and the screen allows a change in the scattering source velocity component along the camera/observer direction. (**B**) Three snapshots acquired by the camera at three different times for an incident angle (θ = 65°) such that the scattering speed toward the camera is subluminal. In this case, the time order is maintained and the perceived source moves from left to right (full video available as Supplementary_Video_1.mp4). (**C**) For θ = 25°, the source velocity toward the detector is superluminal and event time ordering is reversed; that is, the same wavefront measured in (B) is now seen as propagating in the opposite direction, from right to left (full video available as Supplementary_Video_2.mp4). (**D**) The measured speed along the *x* direction (red dots) compared with the theoretical prediction (blue curve).

The experimental setup is shown in Fig. 2A. A time-resolving camera is used to image in-plane scattering from the wavefront generated by a 130-fs laser pulse impinging on an inclined surface (see Materials and Methods). Figure 2B shows a temporal sequence of images taken from the full video (video S1) for the subluminal case (θ = 65°, that is, *v* = *c* cotθ = 0.46*c*). We see the wavefront propagating across the screen from left to right, that is, with the correct temporal ordering of events. Figure 2C shows the same sequence, but for the case in which the scattering event has superluminal speed in the direction of the camera (θ = 25°, that is, *v* = *c* cotθ = 2.14*c*). The wavefront is now seen to propagate in the opposite direction, so temporal ordering is clearly inverted (see also video S2). In Fig. 2D, we compare the measured speed $v_{x}^{0}$ with the prediction of Eq. 2 while systematically increasing the incident angle θ; the results show very good agreement with the predictions.

We stress that the superluminal motion is associated only with the kinematics along the scattering surface; that is, it is not a property of the incoming light pulse itself. The implication of this concept is that the time-ordering inversion that we observe is relative to the scattering surface only and is not an inversion of the local temporal axis of the incoming light pulse. Thus, for example, the time ordering of the plane wave itself (as measured before hitting the screen) is not inverted. This concept is discussed in detail in the Supplementary Materials, where we illustrate that the local time axis of the input pulse is not inverted by the superluminal scattering event, whereas the global time axis is. A possible experimental arrangement to show these effects may rely on wavepackets with strong third-order spectral phase, that is, Airy pulses (the temporal analog of Airy beams) (*13*), in combination with a nonlinear scattering screen.

## IMAGE PAIR CREATION AND ANNIHILATION

Thus far, we have considered the simple case of a flat, tilted scattering screen leading to uniform motion of the source. Interesting effects arise when the source has nonuniform motion, in particular with a subluminal to superluminal transition (or vice versa). Such a situation is obtained by adequately curving the scattering surface. Without loss of generality, we consider the case of a scattering screen described by the function *S*(*x*) = *x*^2^. Here, *x* is a dimensionless quantity corresponding to the spatial coordinate normalized to the length of the screen *L*. Following the very same arguments reported above and considering that in our measurements the screen length was on the order of *L* = 1 m, we find

$$\begin{array}{cc} v_{x}^{0}=\frac{c}{1-2x} & \left(A\right)\\ \frac{dt_{0}}{dt}=1-2x & \left(B\right) \end{array}$$ (3)

Clearly, the perceived speed along the *x* direction is positive for *x* < 0.5 and negative for *x* > 0.5, resulting in two images moving in opposite directions along the *x* axis [from left to right for *x* < 0.5 and from right to left for *x* > 0 (Eq. 3A)]. Correspondingly, the temporal axis at the observation plane is be reversed for *x* > 0.5 (Eq. 3B). As sketched in fig. S3, the observer will therefore see two stripes of light that move toward each other and disappear at *x* = 0.5, a process that we refer to as image pair annihilation. Changing the sign of the curvature of the surface *S*(*x*) will result in the opposite process. Taking, for example, *S*(*x*) = −(*x* − 1)^2^, we have $v_{x}^{o}=c{\left(2x-1\right)}^{-1}$, and the observer will perceive the light wave scattering on the surface as an image pair creation, originating from *x* = 0.5. A similar prediction was recently made by Nemiroff (*14*) albeit in an astronomical setting, for example, in which the curved surface is represented by the edge of the Moon.

**Fig. 3:**
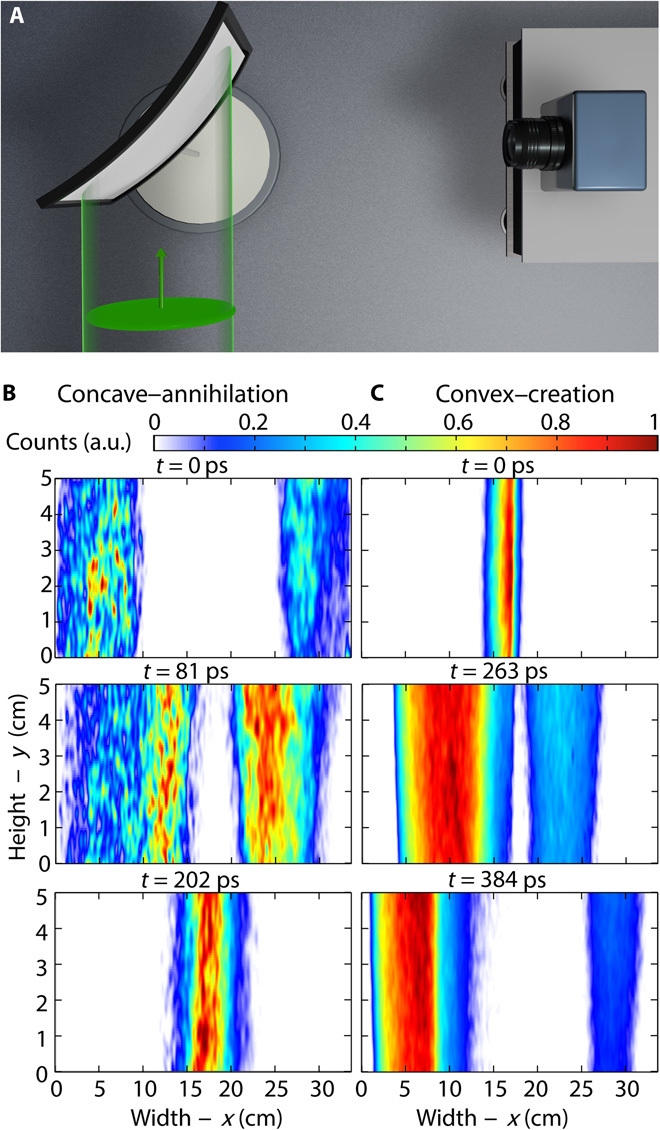
Image pair annihilation and creation. (**A**) Layout of the experiment. (**B**) Three snapshots acquired by the camera at three different times for concave screen, resulting in the annihilation of image pairs (full video available as Supplementary_Video_3.mp4). a.u., arbitrary units. (**C**) Three acquisitions for a convex screen, resulting in the creation of image pairs (full video available as Supplementary_Video_4.mp4).

In Fig. 3, we show an example of an experiment performed using a curved scattering screen (see Fig. 3A) and illustrating the annihilation and generation of image pairs. For a properly chosen concave shape, the camera records the annihilation of an image pair, as shown in the three acquisitions reported in Fig. 3B (see video S3). Similarly, a convex screen results in the creation of an image pair, as shown in Fig. 3C (see video S4). We stress that, at any given time, the propagating wavefront has one and only one intersection point (the scattering source) along any horizontal line on the screen: The observed image splitting is therefore truly a result of the transition between subluminal and superluminal propagation (see the Supplementary Materials).

## DISCUSSION

A crucial element of these experiments is that the geometry allows propagation in the direction of the observer that is faster than the free-space wave propagation speed. Similar image pair effects were predicted in an astronomical context where they might, however, be somewhat harder to observe (*14*). We note here that these effects are not to be confused with the apparent superluminal motion of astrophysical radio sources (*15*) that are due to movement perpendicular to or at an angle with respect to the observer. Here, the observed effects are due to a real superluminality in the direction of the observer.

Similarly to light, the propagation of sound waves or mechanical vibrations may give rise to temporal inversion. Aside from the predictions by Lord Rayleigh, a further example worth considering would be the scattering of seismic waves from an inclined geological surface. The detection of scattered seismic waves is commonly used to determine the composition of the inner layers of the Earth’s structure, yet it is clear from the considerations above that a temporally resolved measurement could give rise to an apparently inverted geophysical structure. Thus, the true kinematics of an event cannot be unambiguously determined by relying solely on imaging and time-resolved measurements. This ambiguity could be removed, for example, by acquiring additional information on the exact conformation of the scattering surfaces, the speed of the source, or the source coordinate along the direction of observation. Such considerations will play a crucial role in emerging time-resolved imaging technologies that rely on detecting scattered light from surfaces (*16*–*21*).

Superluminal motion and its implications have also been widely discussed in a number of contexts such as tachyonic particles and superluminal tunneling (*22*–*26*). In particular, the precise form of the equivalent Lorentz transform for superluminal motion has been widely debated (*22*), with some open problems remaining unresolved (*27*). Experiments such as those shown here could be adapted to provide experimental grounding for the assumptions that underlie such theoretical models.

## MATERIALS AND METHODS

A schematic overview of the experimental layout is shown in Fig. 2A. The illumination source was a 130-fs pulse laser (80-MHz repetition rate, 810-nm wavelength, and 1-W average power) that was diffused to uniformly illuminate the surface of the scattering screen over an area of roughly 50 cm × 50 cm.

The imaging camera was a time-resolving iCCD camera that acquired 520 × 688–pixel images with a 200-ps temporal gate that could be precisely timed to the laser pulse. Enhanced temporal resolution was achieved by coupling this camera with a delay generator with 10-ps temporal resolution (LaVision GmbH). That is, for a fixed laser pulse/gate delay, the laser pulse would appear temporally integrated over the 200-ps duration of the gate. By scanning the gate delay, we created a variable window in time that allowed us to reconstruct the position of the laser wavefront with high temporal resolution. Movies showing the full measurements and detailed evolution of the laser pulse wavefront are provided in the Supplementary Materials, along with supporting data, which were acquired more directly with a SPAD (single-photon avalanche diode) array camera without the scanned-gate method described above.

## Supplementary Material

http://advances.sciencemag.org/cgi/content/full/2/4/e1501691/DC1

## SUPPLEMENTARY MATERIALS

Supplementary material for this article is available at http://advances.sciencemag.org/cgi/content/full/2/4/e1501691/DC1 Supplementary text fig. S1. Layout of the situation described in the text for an angle of observation ϕ independent of the angle of incidence θ. fig. S2. Noninversion of the input pulse time ordering. fig. S3. Superluminal scattering of an optical pulse that changes color in time. fig. S4. SPAD camera measurements. fig. S5. Space-time Minkowski diagrams for image pair creation/annihilation. videos S1 to S4. Reference (*28*)
