# Supplementary material for: Observation of image pair creation and annihilation from superluminal scattering sources
Source: Sci Adv. 2016 Apr 15;2(4):e1501691. doi: 10.1126/sciadv.1501691 (PMC4846444; doi:10.1126/sciadv.1501691)
Supplement: http://advances.sciencemag.org/cgi/content/full/2/4/e1501691/DC1 [file supp_2_4_e1501691__index.html]

Science Advances | Science Advances

## Supplementary Materials

**This PDF file includes:**

- Supplementary text
- fig. S1. Layout of the situation described in the text for an angle of observation  independent of the angle of incidence θ.
- fig. S2. Noninversion of the input pulse time ordering.
- fig. S3. Superluminal scattering of an optical pulse that changes color in time.
- fig. S4. SPAD camera measurements.
- fig. S5. Space-time Minkowski diagrams for image pair creation/annihilation.
- Reference (*28*)

Download PDF

**Other Supplementary Material for this manuscript includes the following:**

- videos S1 to S4 (.avi format).

**Files in this Data Supplement:**

- Adobe PDF - 1501691\_SM.pdf
