# Supplementary material for: Observation of image pair creation and annihilation from superluminal scattering sources
Source: Sci Adv. 2016 Apr 15;2(4):e1501691. doi: 10.1126/sciadv.1501691 (PMC4846444; doi:10.1126/sciadv.1501691)
Supplement: http://advances.sciencemag.org/cgi/content/full/2/4/e1501691/DC1 [file 1501691_SM.pdf]

Science Advances
AAAS

advances.sciencemag.org/cgi/content/full/2/4/e501691/DC1

Supplementary Materials for

Observation of image pair creation and annihilation from superluminal scattering sources

Matteo Clerici, Gabriel C. Spalding, Ryan Warburton, Ashley Lyons, Constantin Aniculaesei,
Joseph M. Richards, Jonathan Leach, Robert Henderson, Daniele Faccio

Published 15 April 2016, *Sci. Adv.* **2**, e501691 (2016)
DOI: 10.1126/sciadv.1501691

This PDF file includes:

- Supplementary text
- fig. S1. Layout of the situation described in the text for an angle of observation ϕ independent of the angle of incidence θ.
- fig. S2. Noninversion of the input pulse time ordering.
- fig. S3. Superluminal scattering of an optical pulse that changes color in time.
- fig. S4. SPAD camera measurements.
- fig. S5. Space-time Minkowski diagrams for image pair creation/annihilation.
- Reference (*28*)

Other Supplementary Material for this manuscript includes the following:
(available at advances.sciencemag.org/cgi/content/full/2/4/e501691/DC1)

- videos S1 to S4 (.avi format).

Supplementary Text

The following document complements discussion in the main text on kinematical effects from superluminal motion of scattering sources. First we provide additional theoretical analysis and consider a more general situation for arbitrary observation and wavefront incidence angle. We then report experimental observations performed for various incident and observation angles, acquired with a different detector (a single photon avalanche photodetector array – SPAD) with respect to that employed in the main text. Finally we expand on the origin of the image pair creation and annihilation effects.

1. General relation for measured velocities

We consider the more general situation depicted in fig. S1, where an incident plane wave with wavevector $\boldsymbol{k}$ scatters from a flat screen tilted in such a way that its normal forms an angle θ with $\boldsymbol{k}$. We first analyse the case of in-plane scattering, where the camera is placed in such a way that the observation direction forms an angle ϕ with the normal to the scattering surface. We wish to evaluate the transformation of the time coordinate into the measured time and the consequent transformation between the speed along the $\boldsymbol{x}'$ direction and the *measured* speed along $\boldsymbol{x}'$. To this end, we start by noting that in the (x, y) reference frame

$$x(t) = c \cdot t$$
$$y(t) = \cot(\theta) \cdot x(t)$$

We can hence evaluate the spatial coordinates of the scattering source in a new reference frame (x', y'), as shown in fig. S1. The angle between the incident wavevector, which defines our x-axis, and the path from screen to detector, which defines the y'-axis, is $(\theta + \phi)$. Thus, the angle between the x- and x'-axes is $(\theta + \phi - \frac{\pi}{2})$, and so the coordinate transformation is given by

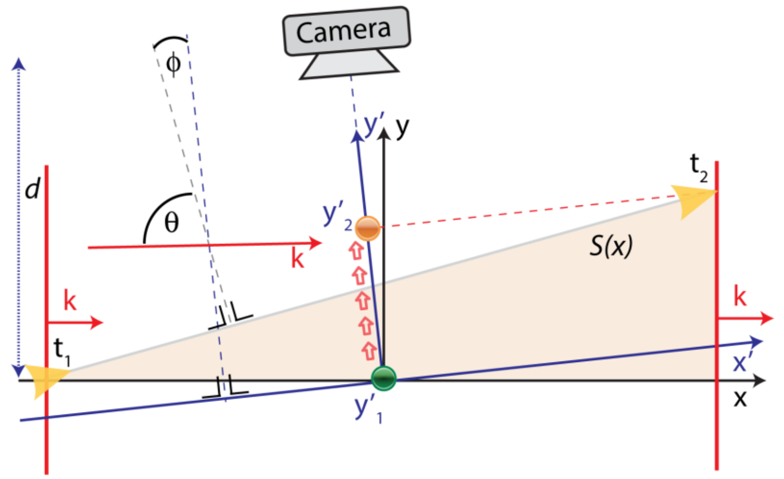

fig. S1. Layout of the situation described in the text, for an angle of observation $\boldsymbol{\phi}$ independent from the angle of incidence $\boldsymbol{\theta}$.

$$x'(x) = \cos\left(\theta + \phi - \frac{\pi}{2}\right) x - \sin\left(\theta + \phi - \frac{\pi}{2}\right) y(x) = x \cos(\phi) \csc(\theta)$$
$$y'(x) = \sin\left(\theta + \phi - \frac{\pi}{2}\right) x + \cos\left(\theta + \phi - \frac{\pi}{2}\right) y(x) = x \csc(\theta) \sin(\phi)\ ,$$

Which leads to

$$x'(x) = \mathrm{c\,t} \cos(\phi) \csc(\theta)$$
$$y'(x) = \mathrm{c\,t} \sin(\phi) \csc(\theta)$$

In this case, the speed along $\boldsymbol{x'}$ and $\boldsymbol{y'}$ can be evaluated as

$$v_{x'} = \frac{dx'}{dt} = \mathrm{c} \cos(\phi) \csc(\theta)$$
$$v_{y'} = \frac{dy'}{dt} = \mathrm{c} \sin(\phi) \csc(\theta) \qquad \text{Eq. 1}$$

In order to find the relation linking the measured time interval t_0 with the original one (t), and the measured speed $v_{x'}^0$ to the actual spot speed relative to the screen $v_{x'}$, we consider that t_0 is the arrival time of the information at the plane of the detector and is obtained by summing two components: t_A, the interval of time over which the scattering source moves from the origin to the point y' and t_B, the time required to the scattered light to propagate in free space from y' to the detector. These read

$$t_A = t$$
$$t_B = t\frac{d - y'(t)}{c}$$

so that $t_0 = t + \frac{d - c\,t \csc(\theta)\sin(\phi)}{c}$, and the relation between the measured and real time is

$$\frac{dt_0}{dt} = 1 - \csc(\theta)\sin(\phi) = 1 - \frac{v_{y'}}{c} \qquad \text{Eq. 2}$$

and the measured velocity along $\boldsymbol{x}'$ reads

$$v_{x'}^{0} = \frac{dx'}{dt_p} = \frac{dx'(t)}{dt}\frac{dt}{dt_0} = \frac{c\cos(\phi)}{\sin(\theta) - \sin(\phi)} \qquad \text{Eq. 3}$$

We note that these equations correctly reduce to those considered in the main text. Indeed for $\phi = {}^{\pi}/_{2} - \theta$, Eq. 2 reduces to

$$\frac{dt_0}{dt} = 1 - \frac{v_y}{c} \qquad \text{Eq. 4}$$

and coincides with Eq. 1 in the text. Similarly, for the same condition Eq. 3 reduces to

$$v_{x'}^{0} = v_x^0 = \frac{c}{1 - \cot(\theta)}$$

which coincides with Eq. 2 in the main text.

2. Non-inversion of the input pulse relative time axis.

When considering the temporal inversion induced by superluminal speed it is important to distinguish the time axis of the superluminal scattering event from the local time axis of the input pulse that is used to create the superluminal scattering source (*e.g.* by impinging at an angle on a screen). Whilst the first is inverted due to the finite speed of light, the latter is not. Figure S2 shows a schematic illustration that explains this point where we consider as an illustrative example, an input pulse composed by an ordered sequence of three bursts of different colours: R-B-G (red, blue and green). In the subluminal speed case (fig. S2A), an observer (the camera in the figure) would see that the scene evolves in time with the original temporal order, *i.e.* the event at the point "-" occurs before that the event at the point "+". Moreover, the colour sequence is the same of the input pulse, *i.e.* R-B-G. In the superluminal speed case on the other hand (fig. S2B), the observer would see the evolution of the superluminal event inverted, as indeed the event in "-" is perceived after that in "+". Yet, the colour order is still the same as that of the input pulse (R-B-G). Indeed, we note that the time ordering identified by the colour sequence R-B-G is not related to a real time axis but rather to the "local" time axis that is associated to the coordinate system co-moving with the scattering optical pulse. We clarify the difference with a second illustrative experiment, shown in fig. S3, where we consider an optical pulse featuring a colour that changes in the laboratory time frame and that scatters on a surface and we consider the superluminal scattering speed case. It is clear from this example that the local time ordering of the input pulse, that changes colour from red to blue to green in propagation, is now mapped onto the time axis of the

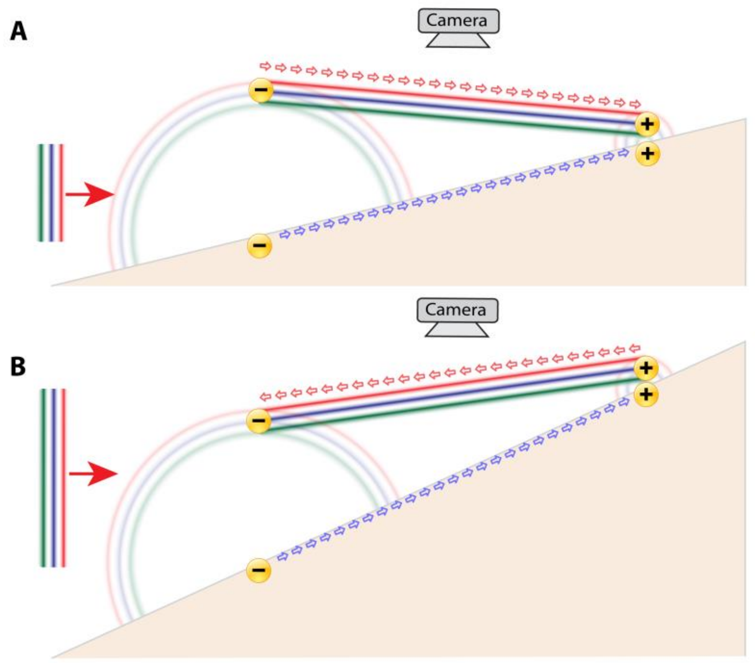

fig. S2. Non inversion of the input pulse time ordering. **A** (**B**) shows the effect of a pulse composed by a red-blue-green sequence, inducing a sub(super)-luminal scattering source. The color ordering perceived by the observer is not inverted in both cases.

scattering source, and is then inverted due to the superluminal scattering speed.
We note however, that this condition is not trivially accessible experimentally. A possible way to observe this effect may rely on wavepackets with strong third order spectral phase, *i.e.* Airy pulses (the temporal analogue of Airy beams) [13], in combination with a nonlinear scattering screen. Indeed, when dispersion is considered, the frequency of the main lobe of an ultrashort Airy pulse changes along propagation [29], *i.e.* the main-lobe frequency is a time-dependent quantity in the laboratory reference frame. Since the main lobe is the most intense part of the optical pulse, this frequency will be the dominant one in a nonlinear (intensity dependent) scattering process, *e.g.* in surface second harmonic generation.

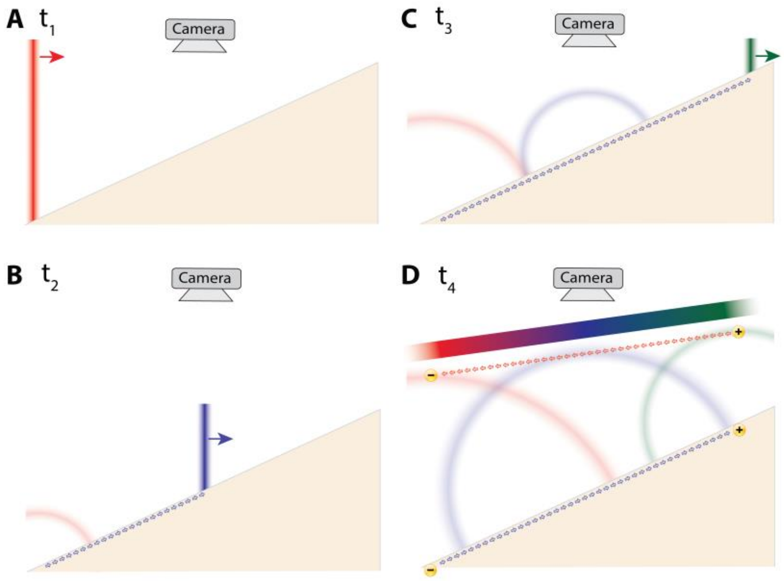

fig. S3. Superluminal scattering of an optical pulse that changes colour in time. **A** shows the pulse scattering at the instant t_1 from the skewed screed (the pulse at t_1 is red). **B** shows the scattering event at t_2, when the pulse is blue. **C** shows the scattering at t_3 (the pulse is green). Finally, **D** shows the scattered signal at a later time t_4. We clearly see that the spherical waves induced by the scattering at t_1, t_2 and t_3 produce a signal that changes colour in time and that is detected by the observer with a color sequence (hence a time sequence) that is opposite to that of the input pulse.

3. Measurements with a SPAD array

To provide independent corroboration of the trends observed via the scanned-gate method we utilized with our intensified CCD camera, we also, more directly, recorded spatially and temporally resolved data with a different detector, namely a 32×32 pixel array of single photon avalanche diodes (SPADs), described in detail in Ref. [18] in the main text, a novel camera with (56 ± 1) ps temporal resolution.

The scattering surface, placed 5 m away was imaged onto the array with a 6-mm focal length lens. The illumination source was the same employed in the experiments described in the main text, however the geometry was different as the condition $\phi = \pi/2 - \theta$ was not satisfied. To provide an example of a more general geometry the angle between the illumination source and the camera direction was set to ~ 55 deg. The screen surface was kept flat but its inclination was changed in order to first have subluminal motion of the plane wave in the direction of the camera ($\theta = 36$ deg, $\phi = 17$ deg) for the data shown in fig. S4A, and then so as to have a superluminal speed ($\theta = 8$ deg, $\phi = 47$ deg), reflected by fig. S4B. From the data acquired with the SPAD camera it is possible to extract the perceived velocity of the image for the two cases. For the first case ($\theta =$

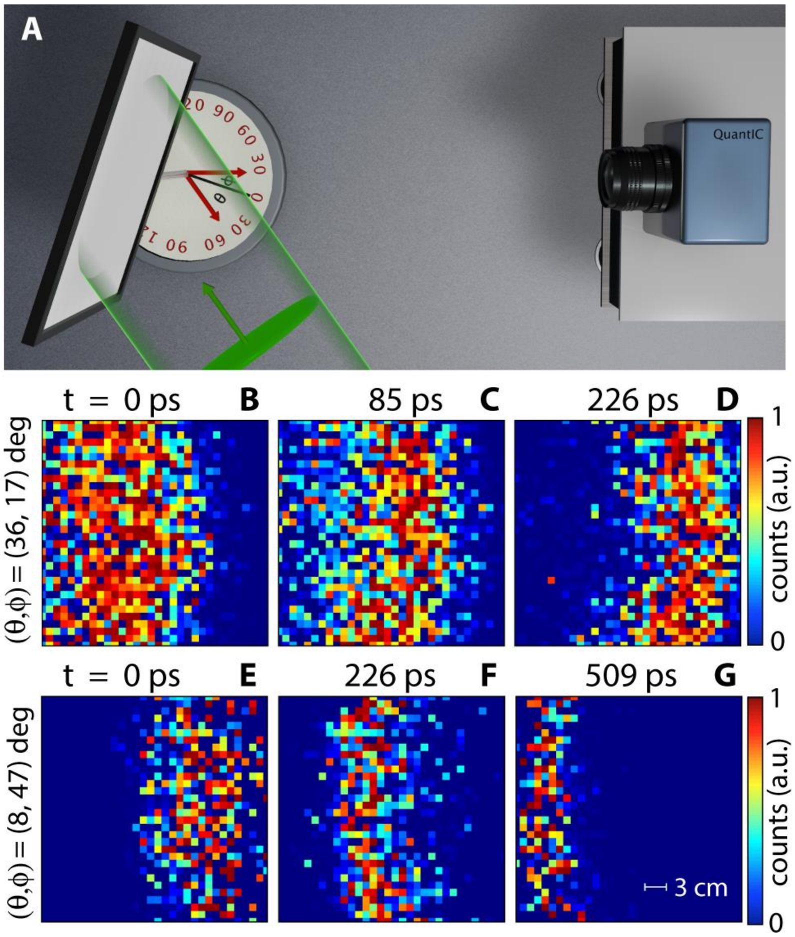

fig. S4. A shows a sketch of the experimental setup employed to perform the time-resolved imaging for the conditions reported above, with a SPAD camera detector. **B-D** show the images recorded for three different instant of time in case of subluminal propagation, whereas **E-G** show the same but for a superluminal scattering source.

36 deg, $\phi = 17$ deg) we found $v^0_{x,1} = (62 \pm 5)$ cm/ns, while for the second case ($\theta = 8$ deg, $\phi = 47$ deg), we found $v^0_{x,2} = (-33 \pm 5)$cm/ns. These values agree qualitatively with predictions obtained from the simple model described above. Indeed, from Eq. 3 it follows that $v^0_{x,1,\text{model}} = (97 \pm 7)$ cm/ns and $v^0_{x,2,\text{model}} = (-35 \pm 2)$ cm/ns. The discrepancy between the measured and predicted values is largely due to a less precise characterization of the interaction geometry and to the relatively coarse temporal resolution offered by direct use of the SPAD camera (60 ps time bins and minimum temporal resolution, to be compared to the enhanced resolution we achieved via 10 ps scan steps in our use of the iCCD camera). Nevertheless, the same effects reported in the in the main text and observed with the iCCD are still clearly visible with the direct approach utilized with the compact SPAD camera.

4. *Pair creation and annihilation*

We comment here on the origin of the splitting in the images observed for curved scattering surfaces. In fig. S5A we show the Minkowski space-time diagram that provides geometrical intuition regarding the measured order of the events for a scattering source moving along a subluminal *curved* trajectory in the $(x - t)$ space. The relation reported in Eq. 4 for a spatial dependent velocity, maps the time to the measured time

$$\frac{dt_0(x)}{dt} = 1 - \frac{v(x)}{c} = 1 - \frac{1}{c}\frac{dx\ (x)}{dt} \qquad \text{Eq. 5}$$

If $\forall\, x, v(x) < c$, the Jacobian in Eq. 5 is always positive, and therefore the relation from t to t_0 is invertible (the Jacobian is nonzero) and the time order is maintained. If $\forall\, x, v(x) > c$, the Jacobian is negative and nonzero, therefore the relation can be inverted and the time order is reversed (see fig. S5B). However, if $\exists\, x_0 : v(x_0) = c$, then the relation is no longer invertible and becomes multiple valued (non-biunivocal). Figure S5C shows an example of such a situation. At point x_1 the local speed passes from subluminal to superluminal and therefore the Jacobian has a zero. The flow of information emanating from spot positions $x_1 < x < x_2$ is mapped onto the observer's time axis coordinates (and is indicated by the procession of blue arrows). We have also mapped the flow of information from spot positions $x_0 < x < x_1$ (indicated by red arrows proceeding from t'_0 to t'_1). Note that information from spot position x_c reaches the camera at the same instant as information from spot position x_2.

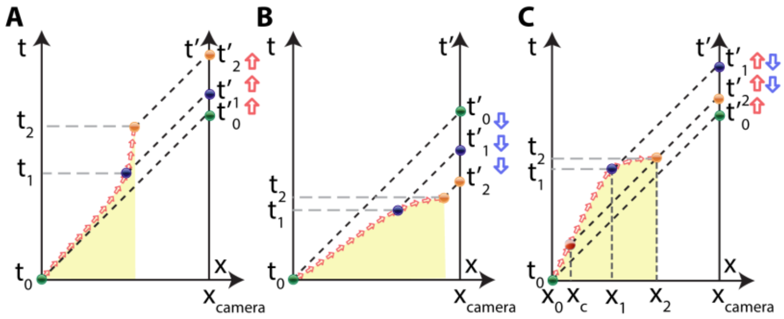

fig. S5. Space-time Minkowski diagrams for image pair creation/annihilation: **A** shows an example of a trajectory of a scattering source along a curved surface that, when imaged, maintains the same time-order for all the points. **B** shows a case of a trajectory that results in a complete time reversal. **C** shows a trajectory that features both behaviours and therefore results in the observation of an image pair (in this specific case, pair annihilation).

At this instant, the camera detects both an image of the spot moving between x_c and x_1, mapped with a positive Jacobian (therefore preserving the order) *and* the image of the spot moving between x_1 and x_2, mapped with a negative Jacobian (therefore inverting the time order). These two images appear to the observer to approach one another until "annihilating" where they meet.
